# Effect of directly acting antivirals for hepatitis C virus infection on proprotein convertase subtilisin/kexin type 9 level

**DOI:** 10.1002/hsr2.273

**Published:** 2021-05-02

**Authors:** Carlo Torti, Vincenzo Scaglione, Bruno Mario Cesana, Chiara Costa, Nadia Marascio, Elisabetta Schiaroli, Chiara Busti, Sabrina Bastianelli, Maria Mazzitelli, Enrico Maria Trecarichi, Daniela Francisci

**Affiliations:** ^1^ Unit of Infectious and Tropical Diseases, Department of Medical and Surgical Sciences “Magna Graecia” University Catanzaro Italy; ^2^ Department of Clinical Sciences and Community Health, Unit of Medical Statistics, Biometrics and Bioinformatics “Giulio A. Maccacaro”, Faculty of Medicine and Surgery University of Milan Milan Italy; ^3^ Unit of Infectious and Tropical Diseases “Mater Domini” Teaching Hospital Catanzaro Italy; ^4^ Unit of Clinical Microbiology, Department of Health Sciences “Magna Graecia” University Catanzaro Italy; ^5^ Infectious Diseases Clinic, Department of Medicine University of Perugia Perugia Italy

**Keywords:** cardiovascular diseases, chronic, hepatitis C, PCSK9, risk

## Abstract

**Background and aims:**

Eradication of the hepatitis C virus (HCV) may affect proprotein convertase subtilisin/kexin type 9 (PCSK9) levels and cardiovascular risk. However, information regarding PCSK9 level after HCV eradication is lacking. Hence, in this case‐control retrospective study, we aimed to evaluate PCSK9 level from pretherapy baseline up to sustained virological response (SVR).

**Methods:**

Eighty‐four patients treated with directly acting antivirals (DAAs) between July 2015 and May 2018 were enrolled. Differences in baseline PCSK9 level due to absence/presence of recorded baseline characteristics (covariates) were evaluated. Changes in PCSK9 levels from pretherapy to SVR (ΔPCSK9) and their correlations with the covariates were assessed. The repeated measures analysis of variance was used to investigate the differences in PCSK9 level from the baseline to the achievement of SVR due to absence/presence of any covariate.

**Results:**

The mean age of the patients was 67.6 ± 11 years, and 53.6% were males. Baseline PCSK9 levels were statistically lower in patients using statins than in those not using statins (mean, 70.3 ± 43.1 ng/mL vs 271.8 ± 252.2 ng/mL; *P* = .017). PCSK9 level decreased significantly from baseline to the time of SVR (255 ± 248 ng/mL vs 169 ± 188 ng/mL; *P* < .001). PCSK9 levels were statistically higher in the HCV‐infected patients at baseline than in the control group (255 ± 248 vs 166.3 ± 120.2 ng/mL; *P* = .020); however, this difference was lost after achieving SVR (mean, 169 ± 188 vs 166.3 ± 120.2 ng/mL; *P* = .464). Changes in PCSK9 level was not statistically related to any of the recorded covariates. The PCSK9 mean level did not differ significantly with absence/presence of any covariate from pretherapy to SVR.

**Conclusions:**

The reduction in mean PCSK9 level from baseline pretherapy to after HCV eradication was statistically significant. Whether PCSK9 is a new biomarker for cardiovascular risk in these patients remains to be ascertained.

## 1 INTRODUCTION

Proprotein convertase subtilisin/kexin type 9 (PCSK9) is an enzyme mainly produced by hepatocytes and encoded by *PCSK9* on chromosome 1 in humans.^1^ Recent studies have elucidated the importance of this molecule in the regulation of liver lipoprotein metabolism.^2^, ^3^, ^4^ In particular, PCSK9 regulates degradation of the low‐density lipoprotein cholesterol receptor (LDL‐R); hence, high levels of PCSK9 are associated with enhanced degradation of LDL‐R in hepatocytes, and therefore, high levels of circulating LDL cholesterol.^5^, ^6^

Syed et al have shown that the hepatitis C virus (HCV) stimulates LDL‐R expression and negatively modulates the expression of PCSK9 in vitro.^7^ In contrast, in vivo studies have demonstrated than PSCK9 levels are higher in HCV‐infected individuals and those coinfected with HIV.^8^, ^9^ High PCSK9 levels predict adverse outcomes in patients with established cardiovascular disease^10^, ^11^ independent of HIV or HCV infection.^8^

A recent study reported increase in PCSK9 level after HCV eradication (ie, sustained virological response, SVR) using combination therapies involving polyethylene glycol (PEG)ylated interferon (IFN).^12^ Another report examining the active and inactive forms of PCSK9 in patients treated with daclatasvir/asunaprevir observed a statistically significant increase in the active form of PCSK9 after SVR, while no statistically significant changes were observed for the inactive form of this molecule.^13^ So far, data regarding the trends in total PCSK9 level after achievement of SVR upon treatment with new regimens, including directly acting antivirals (DAA) without PEG‐IFN, are not available. Owing to paucity of data, the changes in PCSK9 levels in HCV‐infected patients remain unclear.^12^, ^13^

Hence, the main objective of this study was to investigate whether PCSK9 levels changed in patients successfully treated with DAA. We also investigated factors potentially associated with variations in PCSK9 level from baseline (ie, immediately before starting DAA) to the time of achieving SVR.

## 2 METHODS

This analysis was a nested study of the South Italian Network for Rational Guidelines and International Epidemiology (SINERGIE),^14^ which was approved by the Ethics Committee of the Calabria Region (Project identification code #2012.58.E; June 19, 2013). The study was conducted at “Mater Domini” Teaching Hospital of Catanzaro (Southern Italy), in accordance with the guidelines of the Declaration of Helsinki and the principles of good clinical practice.^15^ The patients provided informed consent for participation in the study.

In this case‐control retrospective study, all patients who were treated with DAA between July 1, 2015 and May 31, 2018 were included. Patients who had no pre‐SVR and post‐SVR blood samples available were excluded. A gender‐proportional healthy control group was used for comparison of the PCSK9 levels.

The following data were retrieved at baseline and at the time of SVR from the clinical records: demographics (age and gender), medical history (past and ongoing co‐morbidities), HCV‐RNA viral load, HCV genotype, complete blood count, occurrence of glycaemia, and aspartate aminotransferase (AST), alanine aminotransferase (ALT), gamma‐glutamyl transferase (γGT), creatinine, total cholesterol, total bilirubin, albumin, and α‐fetoprotein levels. All the parameters were measured following internationally standardized methods. HCV RNA viral load was determined using Cobas AmpliPrep/Cobas TaqMan HCV quantitative test v2.0 (Roche Diagnostics, Milan, Italy). Genotyping was performed using Versant HCV genotype v2.0 assay (LiPA, Siemens, Healthcare Diagnostic Inc., Tarrytown, New York). Data regarding the duration and type of DAA, lipid‐lowering agents (ie, statins), and previous treatment with IFN‐based regimens were collected.

Liver fibrosis was estimated either via transient elastography (KPa), or by using the fibrosis‐4 (FIB‐4) score,^16^ or AST to platelet ratio index (APRI).^17^ According to transient elastography, patients were considered cirrhotic when the estimated liver stiffness was ≥14.5 KPa.^18^ Cirrhosis status was excluded or included when APRI index was ≤0.5 or ≥1.5 or when FIB‐4 score was ≤1.45 or ≥3.25. Cases were observed at baseline and after achievement of SVR.

High affinity enzyme‐linked immunosorbent assay (ELISA) (Elabscience) was performed to measure serum PCSK9 levels in cases and control groups in the laboratories of the University of Perugia. The principle of sandwich ELISA was followed. Serum samples were added to the micro ELISA plate and combined with specific antibodies. Then, a biotinylated detection antibodyspecific for PCSK9 was added and the plate was incubated. The unbound components were removed, and a stop solution was added to halt the enzyme‐substrate reactions. Optimal density was measured via spectrophotometry and compared to the standard curve to calculate the PCSK9 levels. The coefficient of variation was <10%. The threshold of detectability of the test was 0.38 ng/mL, and the detection range was 0.63 to 40 ng/mL.

The cases were stratified in subgroups based on the presence or absence of each of the following characteristics (ie, covariates): gender (male vs female), previous use of IFN regimen vs absence of previous IFN treatments, statin co‐medication vs absence of statin comedication, hypertension vs absence of hypertension; liver steatosis (ie, bright liver at ultrasound) vs absence of liver steatosis, dyslipidemia (i.e., total cholesterol >200 mg/dL or use of statin comedications) vs absence of dyslipidemia, diabetes vs absence of diabetes, history of cancer vs absence of history of cancer, and liver cirrhosis vs absence of liver cirrhosis.

### 2.1 Statistical analysis

Differences between cases and control group by gender were assessed using the chi‐square test. The Student's *t*‐test for unpaired data was performed to assess the differences in mean age and mean PCSK9 levels between that at the baseline and at the achievement of SVR in both the cases and control group. The Student's *t‐*test for unpaired data was also performed to assess the differences between the mean PCSK9 levels in the cases (at baseline and at the achievement of SVR) and the control group, and between the PCSK9 mean levels in the case subgroups mentioned above. The repeated measures analysis of variance (ANOVA) was used to investigate the potential differences in PCSK9 expression pattern in cases from the baseline to the achievement of SVR based on absence/presence of covariates, which are expressed as statistically significant interactions called “covariate by time.”

## 3 RESULTS

### 3.1 Patient characteristics

Eighty‐four patients were enrolled and analyzed. The detailed patient characteristics are shown in Table 1. The mean age of the patients was 67.6 ± 11 years, and 53.6% were males. The most common HCV genotype was 1b (59.5%). The most frequent comorbidities were hypertension (50/84; 59.5%), steatosis (33/84; 39.3%), dyslipidemia (16/84; 19%), diabetes (16/84; 19%), previous cancers (18/84; 21.4%), or cirrhosis (33/84; 39.3%). Seven patients (8.3%) were using statins. Twenty‐eight patients (33.3%) had received IFN‐based treatment before starting DAA. The two patients who did not report SVR were excluded from the analysis of the first course of DAA treatment, but were included in the subsequent course after achieving SVR. The following regimens were prescribed: sofosbuvir/daclatasvir in 13/84 patients (15.5%), with addition of ribavirin in one case; sofosbuvir/ledipasvir in 24/84 patients (28.6%), with addition of ribavirin in five cases; sofosbuvir/velpatasvir in 30/84 patients (35.7%); elbasvir/grazoprevir in 17/84 patients (20.2%).

**TABLE 1:** Descriptive statistics of qualitative and quantitative characteristics at baseline of the cohort of HCV infected patients treated with DAAs

Qualitative characteristic	Class	n (%)
Gender	Male	45 (53.6)
Age	≥65 years	57 (67.8)
Genotype	1a	5 (6)
	1b	51 (59.5)
	2	21(25)
	3	4 (4.8)
	4	3 (3.6)
Comorbidities	Hypertension	50 (59.5)
	Hepatic steatosis	33 (39.3)
	Dyslipidaemia	16 (19)
	Diabetes	16 (19)
	Previous cancer	18 (21.4)
	HCC	2 (2.4)
	Cirrhosis	33 (39.3)
Therapeutic history	Naïve	56 (66.6)
	Interferon experienced	28 (33.3)

Quantitative characteristic	Measure unit	Mean (SD)
Age	years	67.6 (11)
Hepatic stiffness	KPa	12.7 (13.4)
HCV‐RNA	IU/mL	2 550 924 (2385381)
PCSK9	ng/mL	255 (248)
Glycaemia	mg/dL	107.8 (21.3)
Total cholesterol	mg/dL	162.4 (37)
Triglycerides	mg/dL	100.3 (56.2)
Albumin	g/dL	4.2 (0.4)
Creatinine	mg/dL	0.9 (0.3)
INR	‐	1.1 (0.1)
AST	U/L	51 (67)
ALT	U/L	56.4 (60.4)
γ‐GT	U/L	49.4 (42.7)
Total bilirubin	mg/dL	0.6 (0.4)
Platelets	10^3^/μL	197 (73.2)
White blood cells	10^3^/μL	6.03 (1.9)
Red blood cells	10^6^/μL	4.62 (0.68)
Haemoglobin	g/dL	13.5 (1.9)
α‐fetoprotein	ng/mL	5.2 (4.4)
APRI Score	‐	0.8 (1.2)
FIB‐4 Score	‐	2.8 (2.4)

Abbreviations: ALT, alanine aminotransferase; APRI, aminotransferase‐platelet ratio index; AST, aspartate aminotransferase; FIB‐4, fibrosis‐4 index; HCC, hepatocellular carcinoma; IFN, interferon; INR, international normalized ratio; γ‐GT, gamma‐glutamyl transferase.

### 3.2 Characteristics of the control group and comparison with cases

Thirty‐eight healthy users were part of the control group. The gender distribution in the control group did not differ significantly from that of the cases: 23/38 (60.5%) individuals were males in the control group vs 45/84 (53.6%) in the cases group (*P* = .513). The mean age of the control group was lower than that in the cases (46.8 ± 13 years vs 67.6 ± 11 years; *P* < .001).

### 3.3 Differences in PCSK9 level at baseline between the patient groups

Table 2 shows baseline PCSK9 levels in the patients' subgroups based on the absence/presence of covariates. Baseline PCSK9 levels were statistically lower in patients using statins than in those not using them (mean 70.3 ± 43.1 vs 271.8 ± 252.2 ng/mL; *P* = .017). Statistically significant borderline differences in PCSK9 levels was observed between females and males (mean 200.7 ± 220.3 vs 302.0 ± 263.1 ng/mL; *P* = .071), and between patients with or without history of cancer (mean 165.8 ± 188.1 vs 279.3 ± 257.8 ng/mL; *P* = .078).

**TABLE 2:** Descriptive statistics of PCSK9 levels at baseline and comparison between the two classes of each covariate

Covariate	Class	Baseline PCSK9 [ng/ml] Mean (SD)	*P*‐value
Gender	Male	302.0 (263.1)	0.071
	Female	200.7 (220.3)	
Previous interferon‐based treatment	Yes	282.9 (254.6)	0.670
	No	241.0 (245.8)	
Statin comedication	Yes	70.3 (43.1)	0.017
	No	271.8 (252.2)	
Hypertension	Yes	207.2 (207.0)	0.140
	No	325.2 (287.3)	
Liver steatosis	Yes	254.9 (264.7)	0.654
	No	255.0 (239.2)	
Dyslipidaemia	Yes	155.6 (178.9)	0.116
	No	278.3 (257.1)	
Diabetes	Yes	171.4 (192.0)	0.131
	No	274.6 (256.7)	
History of cancer	Yes	165.8 (188.1)	0.078
	No	279.3 (257.8)	
Liver cirrhosis	Yes	220.9 (201.6)	0.981
	No	273.0 (269.9)	

*Note: P*‐values are from the unpaired Student's *t‐*test.

### 3.4 Changes in PCSK9 level and possible predictors

**FIGURE 1:**
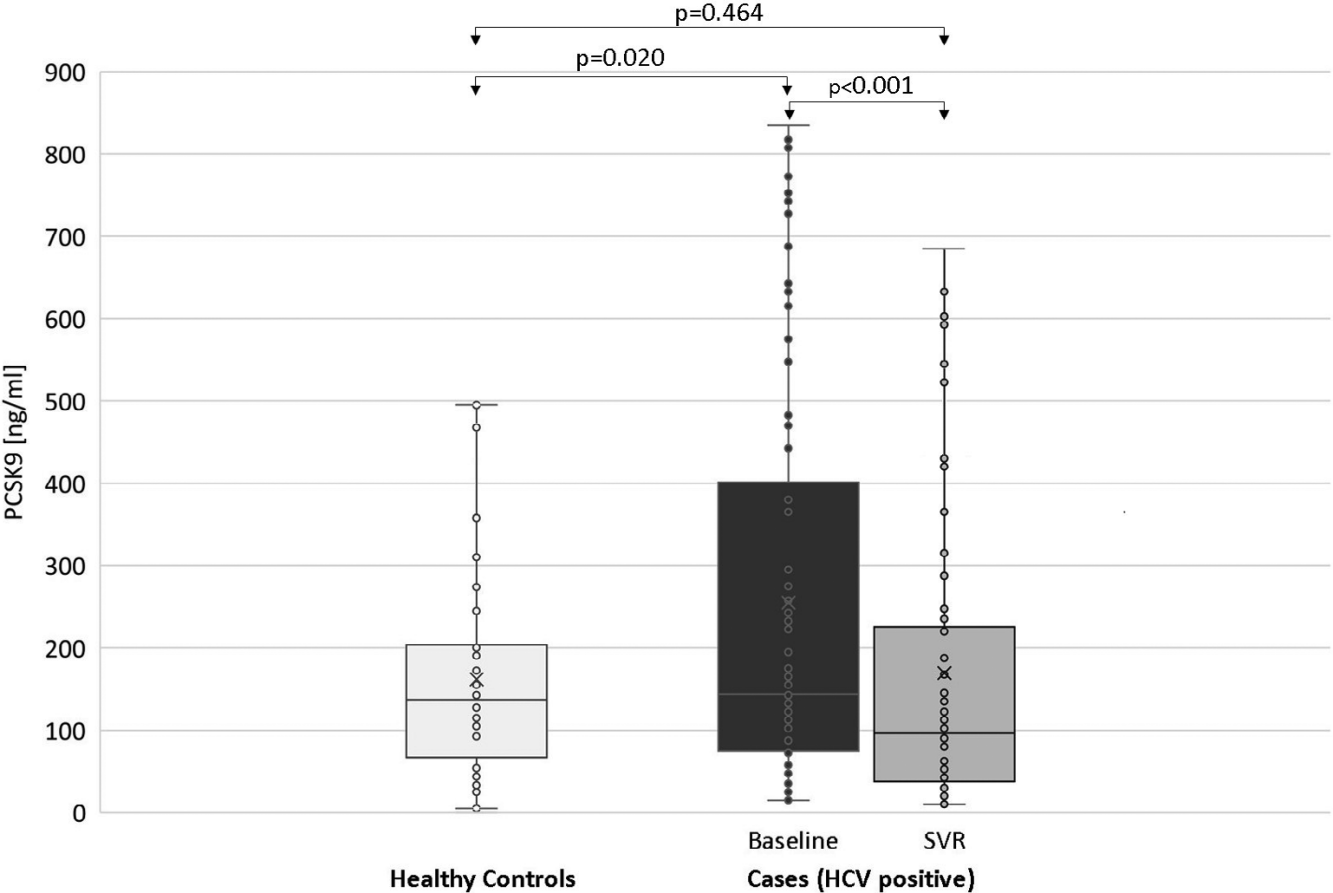
The horizontal lines at the extremities of the vertical segments (whiskers) indicate the minimum and maximum values of PCSK9. Crosses indicate mean values of PCSK9. Boxplots indicate the first and the third quartiles of PCSK9 values (upper and lower sides of the rectangles), and median values (horizontal lines inside the rectangles)

As shown in Figure 1, PCSK9 level decreased significantly from baseline (mean 255 ± 248 ng/mL) to the time of SVR (mean 169 ± 188 ng/mL; *P* < .001), accounting for a mean ΔPCSK9 of 86 ± 112 ng/mL. PCSK9 levels were statistically higher in the HCV‐infected patients at baseline than in the control group (mean 255 ± 248 vs 166.3 ± 120.2 ng/mL; *P* = .020); the difference was not statistically significant when controls were compared with patients after DAA therapy (mean 169 ± 188 vs 166.3 ± 120.2 ng/mL; *P* = .464).

Table 3 shows PCSK9 levels at pretherapy baseline and after achieving SVR. Statistically significant interactions between covariates and time were not detected, indicating that the decrease did not differ significantly with the absence or presence of the covariates considered in the study (see *P*‐values in the same table). The statistically significant difference in the baseline PCSK9 level was maintained until achieving SVR, leading to a parallel decrease in PCSK9 level in patients taking or not taking statins, between male and female patients, and between patients with or without history of cancer.

**TABLE 3:** PCSK9 levels at pretherapy baseline and at achievement of SVR in the classes of covariates

Covariate	Class	Baseline PCSK9 [ng/ml] Mean (SD)	SVR PCSK9 [ng/ml] Mean (SD)	*P*‐value
Gender	Male	302.0 (263.1)	199.2 (204.2)	0.739
	Female	200.7 (220.3)	134.8 (162.4)	
Previous interferon‐based treatment	Yes	282.9 (254.6)	161.5 (188.5)	0.985
	No	241.0 (245.8)	184.9 (188.4)	
Statin comedication	Yes	70.3 (43.1)	49.6 (41.0)	0.678
	No	271.8 (252.2)	180.2 (192.1)	
Hypertension	Yes	207.2 (207.0)	219.6 (220.2)	0.962
	No	325.2 (287.3)	135.1 (155.1)	
Liver steatosis	Yes	254.9 (264.7)	184.3 (207.2)	0.401
	No	255.0 (239.2)	159.6 (175.3)	
Dyslipidaemia	Yes	155.6 (178.9)	111.9 (153.1)	0.759
	No	278.3 (257.1)	182.8 (193.4)	
Diabetes	Yes	171.4 (192.0)	113.6 (165.4)	0.602
	No	274.6 (256.7)	182.4 (191.3)	
History of cancer	Yes	165.8 (188.1)	94.4 (134.7)	0.280
	No	279.3 (257.8)	189.7 (195.6)	
Liver cirrhosis	Yes	220.9 (201.6)	138.4 (166.9)	0.167
	No	273.0 (269.9)	185.6 (197.2)	

*Note: P*‐values are from the interaction “covariate by time” calculated using repeated measures analysis of variance (ANOVA) to investigate the presence of different patterns from pretherapy baseline to SVR between the classes of the covariates.

## 4 DISCUSSION

In this study, changes in PCSK9 levels from pretherapy baseline to achievement of SVR were assessed, showing for the first time, significant reduction in PCSK9 level concomitant with HCV eradication using IFN‐free regimens. Interestingly, although the mean age of the cases was higher than that of the control, the PCSK9 level did not differ significantly from that of the control group after achievement of SVR.

Our observations are comparable with those of Hyrina et al.^12^ However, contrary to our results, they observed a significant increase in PCSK9 level in 27 patients who achieved HCV eradication.^12^ The only other study on PCSK9 level in HCV‐infected individuals^13^ was conducted on patients treated with daclatasvir/asunaprevir, a combination of nonstructural protein inhibitors of NS5A and NS3, which are not available in Europe. Furthermore, in this study,^13^ different laboratory techniques were used to quantify the levels of active and inactive PCSK9. This apparent inconsistency between our results and those of previous studies can be explained as follows.^12^ Hyrina et al's study^12^ was based on only 27 patients. Furthermore, the patients were treated with regimens including PEG‐IFN, which significantly affects biomarkers of inflammation, coagulation, and oxidative stress.^19^ In this context, it is noteworthy that Butt et al^20^ observed higher reduction of cardiovascular disease (CVD) events in DAA‐treated patients than in those treated with IFN‐containing regimens, which complements our observation that DAA treatment results in SVR in HCV‐infected patients and decreases serum levels of PCSK9.

The advent of DAA‐based treatment against HCV has improved life expectancy and the quality of life of patients with chronic infection. The mechanisms of action of these drugs are diverse, targeting nonstructural proteins (NS3, NS4A, NS4B, and NS5A), proteases, and viral (HCV) polymerase (NS5B).^21^ Current guidelines recommend treatment of all patients with chronic HCV infection using DAA regimens minus IFN.^22^

SVR, corresponding to HCV eradication, is obtained in more than 95% of the treated patients for most DAA‐containing regimens in clinical trials,^23^ which has also been confirmed in real‐life studies including our cohort from which patients were selected for the present analysis based on availability of serum samples taken before and after DAA therapy.^24^ However, some major clinical adverse events, such as arrhythmia, impairment of cardiac function, or increased prevalence of dyslipidemia have been reported.^25^, ^26^, ^27^ In contrast, several studies have shown the clinical benefit of SVR induced by DAA treatment with respect to “hard” clinical endpoints such as CVD‐related mortality.^20^, ^28^, ^29^

Hence, although DAA regimens are associated with worsening of serum lipid profile^27^ and, albeit rarely, with cardiac toxicity (ie, arrhythmia),^25^, ^26^ SVR achievement is associated with significant improvement of cardiovascular status.^20^, ^28^, ^29^ Therefore, our results are interesting, as they may explain the reduction in CVD events and atherosclerosis after DAA‐mediated HCV eradication.

However, our study has some limitations. The reduction in PCSK9 level should not be interpreted simplistically. Our results show large standard deviations in PCSK9 levels in the cases and the control group; the considerable variation in the levels of this molecule in the samples studied may be explained by the complex relationships between several variables, which are actually not completely understood and not well investigated owing to the small size of our sample. Furthermore, the control group was proportionally homogeneous with respect to gender, but not regarding age, which is because of the prevalence of the HCV infection in aged people in Italy.^24^ Finally, the efficacy of different DAA regimens targeting related virus molecules (ie, first and second‐generation protease inhibitors and nucleoside or nonnucleoside inhibitors of viral polymerase) or the significance of using ribavirin as part of the therapeutic regimen were not explored due to the limited sample size. Further studies are required to investigate in detail PCSK9 levels in HCV‐infected patients and the factors regulating the levels of this molecule.

## 5 CONCLUSIONS

In conclusion, we observed for the first time that PCSK9 level may decrease concomitantly with SVR induced by DAA therapy, and that this pattern of decrease was consistent independent of the absence or presence of any of the covariates analyzed. Our observations are consistent with, and possibly explain, the reduction in CVD observed in clinical studies.^20^, ^28^, ^29^ It remains to be determined whether PCSK9 predicts cardiovascular risk in HCV‐infected patients.

## CONFLICT OF INTEREST

Nothing to declare.

## AUTHOR CONTRIBUTION

Conceptualization: Carlo Torti, Bruno Mario Cesana, Enrico Maria Trecarichi, Daniela Francisci.

Data curation: Vincenzo Scaglione, Chiara Costa, Nadia Marascio, Elisabetta Schiaroli, Chiara Busti, Sabrina Bastianelli, Maria Mazzitelli.

Formal analysis: Bruno Mario Cesana, Daniela Francisci.

Investigation: Vincenzo Scaglione, Elisabetta Schiaroli, Chiara Busti, Sabrina Bastianelli, Daniela Francisci.

Methodology: Bruno Mario Cesana, Daniela Francisci.

Project administration: Carlo Torti.

Supervision: Bruno Mario Cesana, Enrico Maria Trecarichi, Daniela Francisci.

Validation: Carlo Torti, Bruno Mario Cesana.

Writing ‐ original draft: Carlo Torti, Vincenzo Scaglione, Bruno Mario Cesana.

Writing ‐ review and editing: Chiara Costa, Nadia Marascio, Elisabetta Schiaroli, Chiara Busti, Sabrina Bastianelli, Maria Mazzitelli, Enrico Maria Trecarichi, Daniela Francisci.

## TRANSPARENCY STATEMENT

None.

## ETHICS STATEMENT

All subjects provided informed consent for participation in to the study. The study was conducted in accordance with the Declaration of Helsinki, and the protocol was approved by the Ethics Committee of Calabria Region (Project identification code #2012.58.E; June 19, 2013).

## Data Availability

The data that support the findings of this study are available on request from the corresponding author. The data are not publicly available due to privacy or ethical restrictions.
